# Gentamicin promoted the production of CD4^+^CD25^+^ Tregs via the STAT5 signaling pathway in mice sepsis

**DOI:** 10.1186/s12865-022-00521-4

**Published:** 2022-09-26

**Authors:** Jinfeng Li, Fengdan Xu, Song Li, Mingyu Xie, Ning Li

**Affiliations:** Department of Neonatology, Guangdong Medical University Affiliated Dongguan Children’s Hospital, No. 68 Xi Hu Third Road, Shilong Town, Dongguan, 523325 Guangdong China

**Keywords:** Neonatal sepsis, Regulatory T cell, Gentamicin, STAT5, Inflammation

## Abstract

**Background:**

Increasing studies have reported that gentamicin (GNT) plays an essential role in sepsis; however, its underlying mechanism is still unclear. In this study, we investigated the mechanism of GNT in sepsis.

**Results:**

We observed that GNT enhanced survival and alleviated inflammatory injuries of the lungs, liver, kidneys, and intestines in mice with sepsis. Furthermore, regulatory T cells (Tregs) showed enhanced inhibitory function, and pro-inflammatory cytokines IL-1β, TNF-α, and IL-2 and anti-inflammatory cytokine IL-10 showed decreased and increased peritoneal fluid levels, respectively, after treatment with GNT. GNT showed enhanced phosphorylation of signal transducer and activator of transcription 5 (p-STAT5) in Tregs in vivo and in vitro. The STAT5 inhibitor restrained the increased functional changes of Tregs and reduced inflammatory responses induced by GNT in vitro. Moreover, the STAT5 inhibitor reversed GNT-mediated impacts on survival and inflammation, and the percentage, apoptosis, and phenotypic and functional changes of Tregs in neonatal sepsis.

**Conclusions:**

Our study revealed that GNT regulates the function of Tregs via the STAT5 signaling pathway, alleviating inflammatory injuries, and provides novel evidence in the treatment of neonatal sepsis.

**Supplementary Information:**

The online version contains supplementary material available at 10.1186/s12865-022-00521-4.

## Background

Neonatal sepsis is an organ dysfunction syndrome caused by bacterial, viral, or fungal infection and is diagnosed in infants less than 28 days old. Neonatal sepsis is identified by imbalances observed in the circulatory shock, inflammatory response, immune dysfunction, and multisystem organ failure [1, 2]. Sepsis is one of the major causes of newborn deaths worldwide. A study reported that sepsis caused 16% of approximately 421,000 newborn deaths worldwide [3]. Another study revealed that among every 1000 newborns, 9.5 showed culture-positive sepsis [4]. Although there have been significant advances in critical care medicine, the morbidity and mortality rates of neonatal sepsis remain high [5]. Therefore, an increased understanding of the molecular mechanisms of sepsis is essential to develop new therapeutic strategies for its treatment.

The immune responses in sepsis are characterized by hyperinflammation in early-onset sepsis and hypoinflammation in late-onset sepsis [6]. CD4^+^CD25^+^ regulatory T cells (Tregs), derived from the lymphatic system, mediate immunosuppression and immune response, maintaining an immune balance [7]. Increasing pieces of evidence have reported that Tregs play a central role in immunosuppression and controlling inflammation in sepsis [8, 9]. Moreover, CD4^+^CD25^+^ Tregs significantly increase the early-onset sepsis, aiding in the early diagnosis of sepsis [10]. In addition, the apoptotic rate of Tregs is lower in sepsis [11]. Thus, Tregs-based therapy offers a novel insight into sepsis.

The signal transducer and activator of transcription 5 (STAT5) is expressed in all lymphocytes and is fundamental to the mammalian immune system [12]. Phosphorylated STAT5 (p-STAT5) leads to dimerization, nuclear translocation, and DNA core motif binding to modulate target gene transcription [13]. Further, it accelerates the proliferation and differentiation of Tregs [14], leading to more inflammatory cytokines [15]. Hence, STAT5 plays a crucial role in lymphocyte development and function. However, the exact molecular mechanism of STAT5 remains unclear.

Gentamicin (GNT) is a broad-spectrum aminoglycoside antibiotic with bactericidal effects on gram-negative and gram-positive bacteria. GNT is frequently used as an anti-infective therapy in patients with sepsis [16, 17]. Several studies have shown that antibiotics can regulate the proliferation and differentiation of Tregs, which modulated the inflammatory cytokines [18–21]. However, it is unclear whether the effect of GNT on neonatal sepsis is associated with Tregs and its molecular mechanism.

In the present study, GNT improved the survival and reduced the inflammatory responses of lipopolysaccharide (LPS)-induced neonatal sepsis. Furthermore, GNT enhanced the functional changes of Tregs induced by sepsis and upregulated p-STAT5 expressions both in vivo and in vitro. The STAT5 inhibitor suppressed the GNT-induced functional changes of Tregs and decreased the inflammatory responses in vitro. And the STAT5 inhibitor rescued GNT-mediated impacts on survival and inflammation, and the percentage, apoptosis, and phenotypic and functional changes of Tregs in neonatal sepsis. Therefore, our results indicated that GNT could mitigate inflammatory damage by regulating the function of Tregs via the STAT5 signaling pathway. Our study not only understands the role of GNT in neonatal sepsis but also provides novel insight into its treatment.

## Materials and methods

### Animal experiment

All animal experiments were approved by the Institutional Animal Care and Use Committee at the University of Guangdong Medical University Affiliated Dongguan Children’s Hospital. Newborn C57BL/6J mice (5–7 days-old) were obtained from the Laboratory Animal Center of Southern Medical University (Protocol Number: GDY2002334). All mice were randomly assigned to four experimental groups (n = 10): control group (mice intraperitoneally injected with saline and intramuscularly injected with saline for 7 consecutive days); GNT group (mice intraperitoneally injected with saline and intramuscularly injected with 7.5 mg/kg/day GNT for 7 consecutive days); LPS group (mice intraperitoneally injected with 10 mg/kg LPS and intramuscularly injected with saline for 7 consecutive days); LPS + GNT group (mice intraperitoneally injected with 10 mg/kg LPS and intramuscularly injected with 7.5 mg/kg/day GNT for 7 consecutive days); LPS + GNT + pimozide group (mice intraperitoneally injected with 10 mg/kg LPS, and intramuscularly injected with 7.5 mg/kg/day GNT and intraperitoneally injected with 1 mg/kg/day STAT5 inhibitor (pimozide) for 7 consecutive days), and LPS + GNT + STAT5 inhibitor (STAT5-IN-1) group (mice intraperitoneally injected with 10 mg/kg LPS, and intramuscularly injected with 7.5 mg/kg/day GNT and orally given 5 mg/kg/day STAT5-IN-1 for 7 consecutive days). The observational experiment was performed on survival mice for 7 days. The mice were then euthanized with carbon dioxide after 7 days. The lungs, liver, kidneys, and intestines of mice were excised and collected for hematoxylin and eosin (HE) staining, and the peritoneal fluids of mice were collected for ELISA assay.

### CD4^+^ T lymphocytes, CD4^+^CD25^+^ Tregs isolation, cell culture, and stimulation

Mouse spleen was treated using collagenase D. Mononuclear cells were obtained using density gradient centrifugation. The CD4^+^ T lymphocytes and CD4^+^CD25^+^ Tregs were isolated from mononuclear cells using mouse CD4^+^ T cell and CD4^+^CD25^+^ Treg cell isolation kits, respectively (Miltenyi Biotec GmbH, Bergisch Gladbach, NRW, Germany). All procedures followed the manufacturers’ instructions. For detection of %CD4^+^CD25^+^cells among CD4^+^ T lymphocytes, the antibodies (PE-anti-CD25 and FITC-anti-CD4) were analyzed by two-color flow cytometry. To stimulate the production of inflammatory cytokines using CD4^+^CD25^−^ T cells, CD4^+^CD25^+^ Tregs or CD4^+^CD25^−^ T cells were treated with anti-CD3 (1 mg/mL) and anti-CD28 (1 mg/mL) antibodies for polyclonal activation of CD4^+^CD25^−^ T cells. All cells were maintained at 37 °C in 5% CO_2_ in RPMI 1640 medium (Invitrogen, Carlsbad, CA, USA) supplemented with 10% fetal bovine serum (FBS, Sigma Aldrich, St. Louis, MO, USA), 100 U/mL penicillin/streptomycin, and 200 ng/mL L-glutamine.

For the culture experiments, we divided cells into six groups: (1) control group (medium), (2) GNT group (750 μM), (3) LPS group (0.2 mg/mL), (4) LPS + GNT group (750 μM GNT + 0.2 mg/mL LPS), (5) LPS + GNT + STAT5 inhibitor (pimozide) group (750 μM GNT + 0.2 mg/mL LPS + 10 µM pimozide), (6) LPS + GNT + STAT5-IN-1 group (750 μM GNT + 0.2 mg/mL LPS + 10 μM STAT5-IN-1). 24 h after treatment, cell culture supernatants were collected for further experiments.

### Quantitative real-time reverse transcription polymerase chain reaction (qRT-PCR)

Total RNA from Tregs was isolated using TRIzol kit (Invitrogen) according to the manufacturer’s instructions. Total RNA was reverse transcribed to cDNA using PrimerScript RT Master Mix (Takara, Dalian, China). qPCR was performed to examine forkhead helix transcription factor p3 (FOXP3) and cytotoxic T-lymphocyte-associated antigen 4 (CTLA-4) levels on 7300 PCR System (Applied Biosystems, Foster City, CA, USA). Relative expression levels were quantified in triplicates and according to the 2^−ΔΔCt^ method. β-actin served as the endogenous control. Table 1 shows the primer sequences used in this study.

**Table 1:** The sequences of primers used in this study

β-actin-F	TCAGGGAGTAATGGTTGGAAT
β-actin-R	GGTCTCAAACATAATCTGGGTCA
FOXP3-F	CAACAACTCAGTCCCGCCT
FOXP3-R	TCGGACACAAAGGAACTGC
CTLA4-F	TGTCACAGGGCTCAGTTGC
CTLA4-R	CTCAGGAATGAGGCATTTC

### Western blot

Total protein was extracted from Tregs using RIPA lysis buffer (Beyotime Biotechnology, Shanghai, China) and quantified using a BCA protein assay kit (Beyotime Biotechnology). In total, 30 µg of total proteins were separated using 10% SDS-PAGE and transferred to a PVDF membrane. After blocked, the membranes were incubated overnight at 4 °C with primary antibodies, including p-STAT antibody (1:1000, Cell Signaling Technology, Beverly, MA, USA), STAT antibody (1:1000; Cell Signaling Technology), FOXP3 antibody (1:1000; Abcam, Cambridge, MA, USA), CTLA-4 antibody (1:1000; Abcam), and GAPDH antibody (1:5000; Abcam), followed by secondary antibody (1:5000; Abcam) for 1 h. The protein bands were detected using enhanced chemiluminescence. Finally, the protein expression levels were quantified using ImageJ software and normalized to GAPDH.

### HE staining

Paraffin-embedded lung, liver, kidney, and intestine tissues were sectioned into 4 μm-thick blocks. Tissue sections were dewaxed, rehydrated, and stained with hematoxylin for 5–10 min. Subsequently, the tissue sections were rinsed with tap water and blued using 1% aqueous hydrochloric acid. After the tissue sections were stained with eosin for 1–2 min, they were observed under the microscope. Morphological changes of lung tissues were scored as minimum (0), mild (1), moderate (2), severe (3), maximum (4) injury based on the presence of alveolar hemorrhage, infiltration or aggregation of inflammatory cells and thickness of the alveolar wall, ranging from 0 to 12 [22]. Liver and kidney sections were scored on inflammation, necrosis/abscess formation, and thrombus formation using the scale given above with a maximum score of 12 [23, 24]. Intestine sections were scored on congestion, intestinal wall thickening, ulcer, and adhesion using the scale given above with a maximum score of 16 [25].

### Flow cytometry apoptosis assay

Tregs were digested using trypsin and centrifuged for 5 min. After Tregs were resuspended, they were stained in Annexin V-FITC/PI (BD Biosciences, San Diego, CA, USA) for 30 min. Finally, the apoptotic rate was analyzed using flow cytometry (BD Biosciences).

### Enzyme-linked immunosorbent assay (ELISA)

The levels of TNF-α, IL-2, IL-10, and IL-1Β in the peritoneal fluid were measured using ELISA kits (R&D Systems, Minneapolis, MN, USA) according to the manufacturer’s protocols.

### Statistical analysis

All data were analyzed using SPSS 22.0 statistical software (IBM, Armonk, NY, USA). These data were expressed as mean ± standard deviation (SD). Comparisons of measurement data among multiple groups were performed using one-way ANOVA test. *P* < 0.05 was considered as a significant difference.

## Results

### GNT improved survival and reduced tissue inflammatory responses in LPS-induced neonatal sepsis

**Fig. 1:**
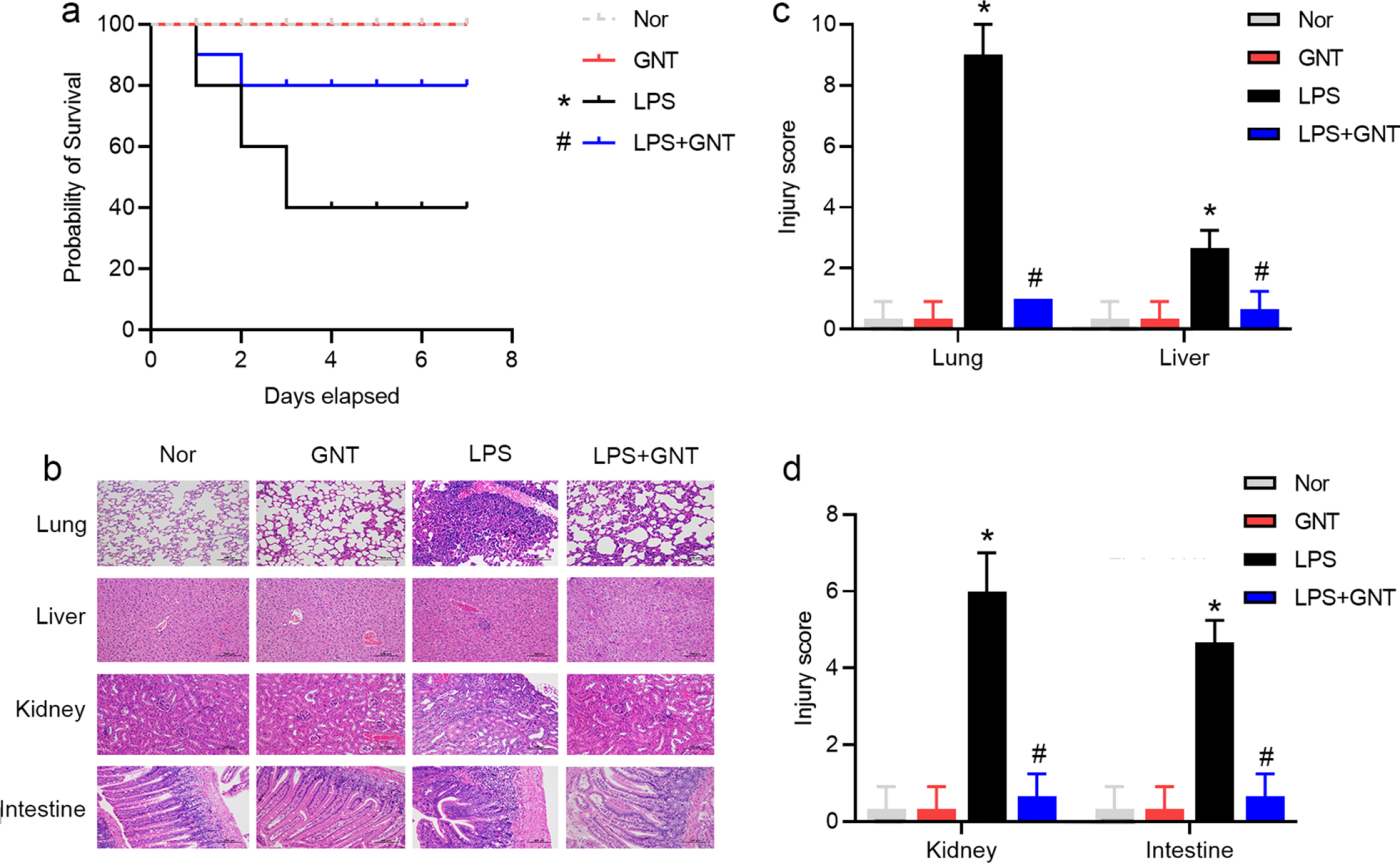
GNT improved survival and reduced tissue inflammatory responses in LPS-induced neonatal sepsis. **a** GNT increased the survival outcome in LPS-induced neonatal sepsis. The survival was monitored for up to 7 days. **b** Tissues from each group were processed for histopathological examination. Newborn mice treated with LPS were highly susceptible to inflammation, neutrophil infiltration, and tissue injury in the lungs, liver, kidneys, and intestines. GNT attenuated these changes in the LPS group. **c** The degree of lung, liver, kidney, and intestine damage was determined using lung, liver, kidney, and intestine injury scoring. Data are expressed as mean ± SD, n = 10. *P < 0.05 versus control group; ^#^*P* < 0.05 versus LPS group

To explore the effect of GNT on the survival rate of mice with LPS-induced neonatal sepsis, we monitored the survival rate of mice after LPS with or without GNT for 7 days. Based on the Kaplan–Meier survival curves, GNT pretreatment prolonged the survival rate of mice with LPS-induced neonatal sepsis. However, GNT treatment alone did not affect the survival rate (Fig. 1a). Next, we evaluated the inflammatory tissue injuries through histological observation. In the control and GNT alone groups, lung, liver, kidney, and intestinal tissues showed normal structures. However, in the LPS group, lung, liver, and intestinal tissues showed increased inflammatory infiltration activity, exhibiting inflammatory cell infiltration. The brush boundary of the renal tissue disappeared obviously, and the tubule lumen dilated slightly. In the GNT + LPS group, the histopathology changes of lungs, liver, kidneys, and intestines were alleviated compared to those in the LPS group (Fig. 1b, c). These results suggested that GNT could increase the survival outcome and attenuate the tissue injuries in mice with neonatal sepsis.

### Effect of GNT on LPS-induced production of inflammatory cytokines

**Fig. 2:**
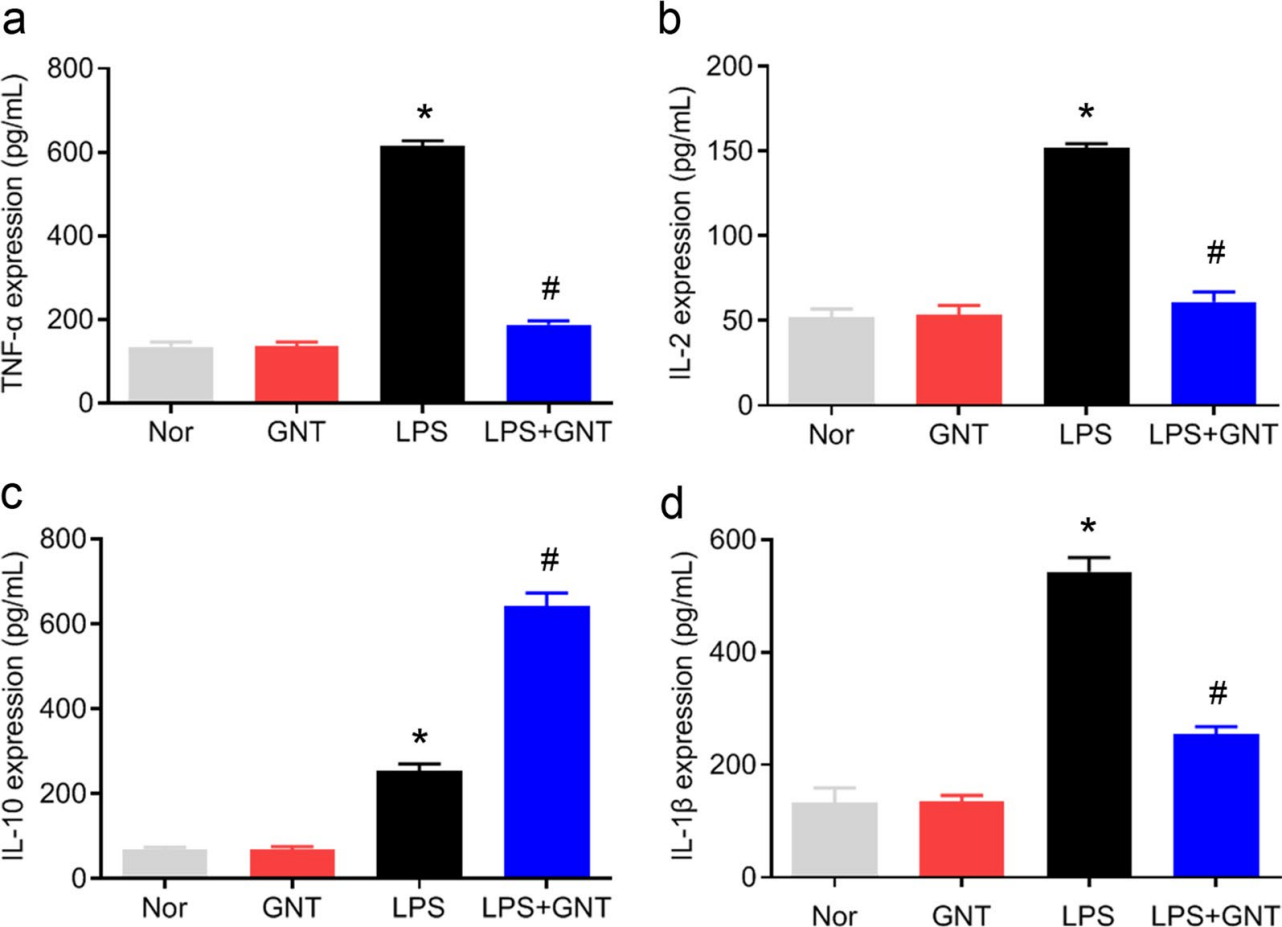
Effect of GNT on LPS-induced production of inflammatory cytokines. Mice with sepsis were treated with or without GNT, and peritoneal fluid was extracted. Inflammatory cytokines TNF-α (**a**), IL-2 (**b**), IL-10 (**c)**, and IL-1β (**d**) were measured using ELISA. Data are expressed as mean ± SD, n = 10. *P < 0.05 versus control group; ^#^*P* < 0.05 versus LPS group

To investigate the anti-inflammatory effect of GNT in LPS-induced neonatal sepsis, the concentrations of inflammatory cytokines were measured using ELISA. Figure 2 shows that the concentrations of pro-inflammatory cytokines TNF-α, IL-2, and IL-1β, and anti-inflammatory cytokines IL-10 in the peritoneal fluid of mice in the LPS group increased compared to that in the control and GNT alone groups. GNT treatment decreased LPS-induced elevation of TNF-α, IL-2, and IL-1β expression levels and increased LPS-induced elevation of IL-10 expression levels in the peritoneal fluid, indicating the anti-inflammatory effect of GNT in neonatal sepsis.

### GNT increased the percentages of CD4^+^ Tregs and decreased the apoptotic rate of CD4^+^ Tregs in neonatal sepsis

**Fig. 3:**
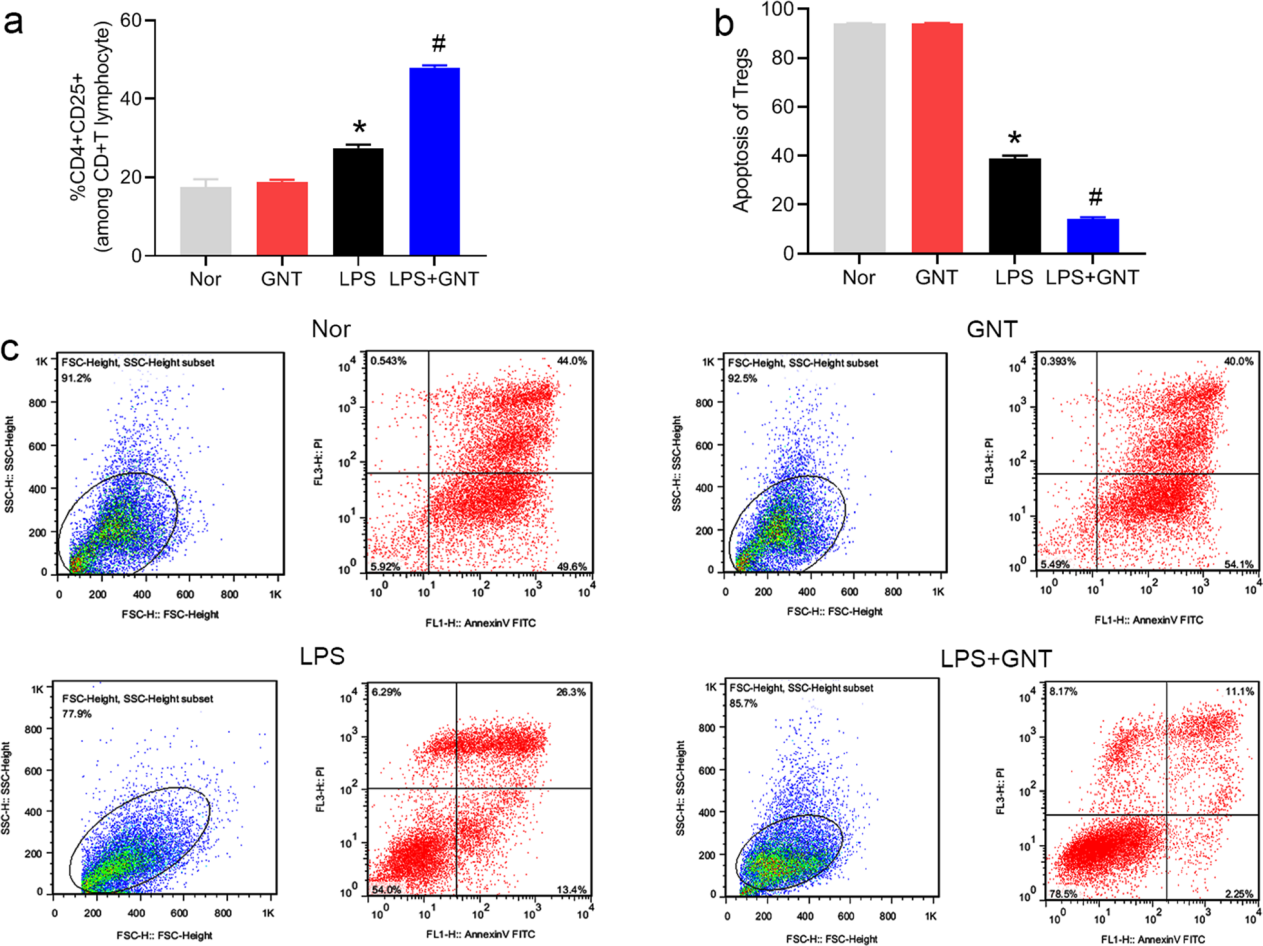
GNT increased the percentages of CD4^+^ Tregs and decreased the apoptotic rate of CD4^+^ Tregs in neonatal sepsis. Percentage (**a**) and apoptotic rate (**b**) of CD4^+^ Tregs in mice with LPS-induced neonatal sepsis treated with or without GNT. **c** Representative images of apoptotic Tregs generated using flow cytometry. Data are expressed as mean ± SD, n = 10. *P < 0.05 versus control group; ^#^*P* < 0.05 versus LPS group

To evaluate the effect of GNT on the quantity of Tregs in mice with sepsis, the percentage and apoptotic rate of CD4^+^ Tregs were examined. As shown in Fig. 3a, Additional file 1: Fig. S1b, LPS dramatically increased the percentage of CD4^+^ Tregs, and GNT aggravated LPS-increased percentage of CD4 + Tregs. Moreover, GNT aggravated the decrease in the apoptotic rate of CD4^+^ Tregs induced by LPS (Fig. 3b, c).

### GNT promoted phenotypic and functional changes of Tregs in neonatal sepsis

**Fig. 4:**
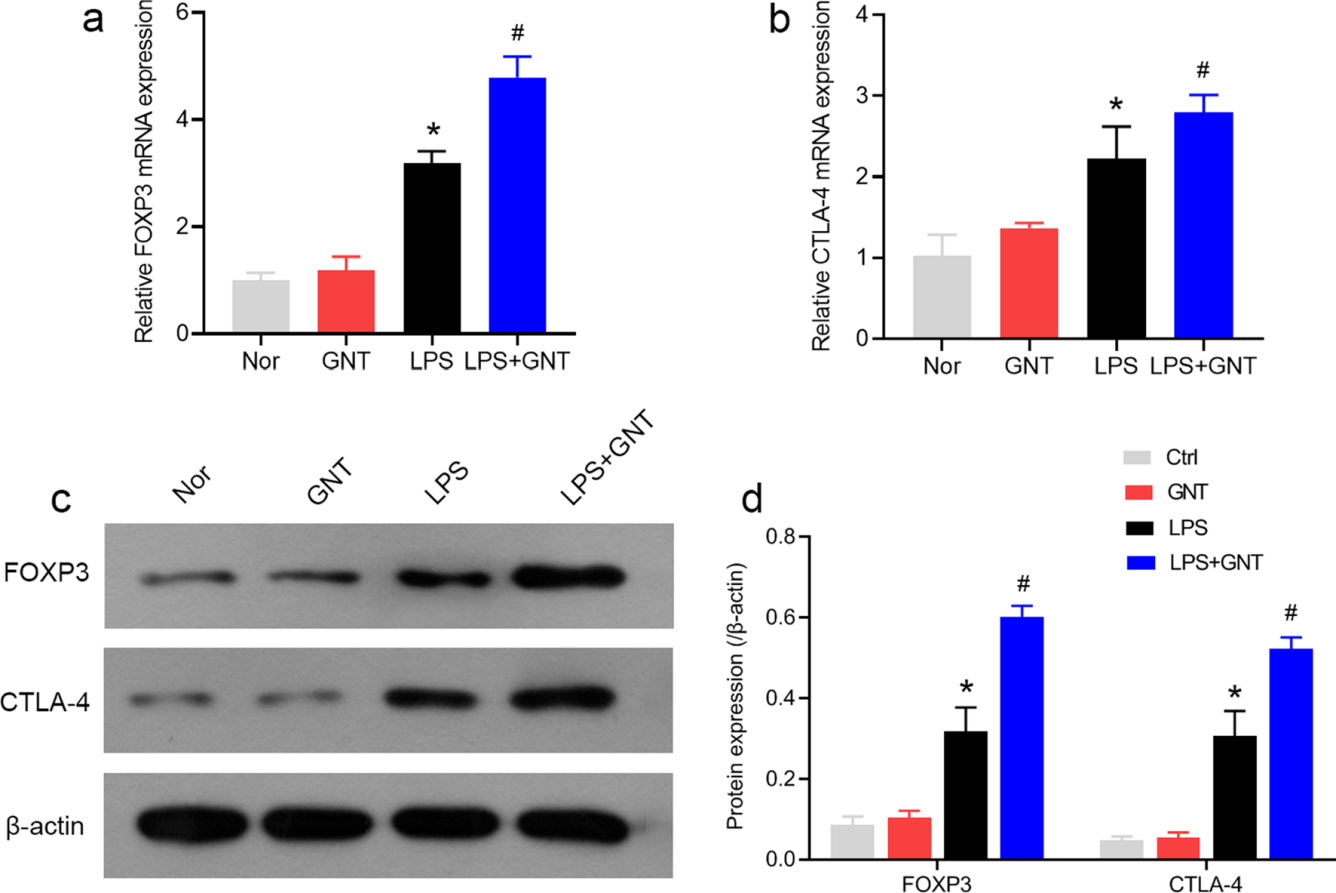
GNT promoted phenotypic and functional changes of Tregs in neonatal sepsis. FOXP3 (**a**) and CTLA-4 (**b**) mRNA expression levels in Tregs were measured using qRT-PCR. Data are expressed as mean ± SD, n = 10. **c**, **d** FOXP3 and CTLA-4 protein expression levels were examined using western blot. Data are expressed as mean ± SD, n = 3. *P < 0.05 versus control group; #*P* < 0.05 versus LPS group

We explored the potential effect of GNT on LPS-induced phenotypic and functional changes of Tregs. The expressions of FOXP3 and CTLA-4 were analyzed using qRT-PCR and western blot analysis. As shown in Fig. 4a, b, LPS significantly upregulated FOXP3 and CTLA-4 mRNA expression levels compared to that with the control and GNT alone groups, and GNT further facilitated LPS-increased FOXP3 and CTLA-4 mRNA expression levels. Furthermore, FOXP3 and CTLA-4 protein expression levels in Tregs were significantly increased in the LPS group compared to that in the control and GNT alone groups, and GNT further enhanced these effects (Fig. 4c, d) (Additional file 2: Fig. S2, Additional file 3: Fig. S3).

### GNT modulated the phenotypic and functional changes of Tregs in vitro via activation of the STAT5 signaling pathway

**Fig. 5:**
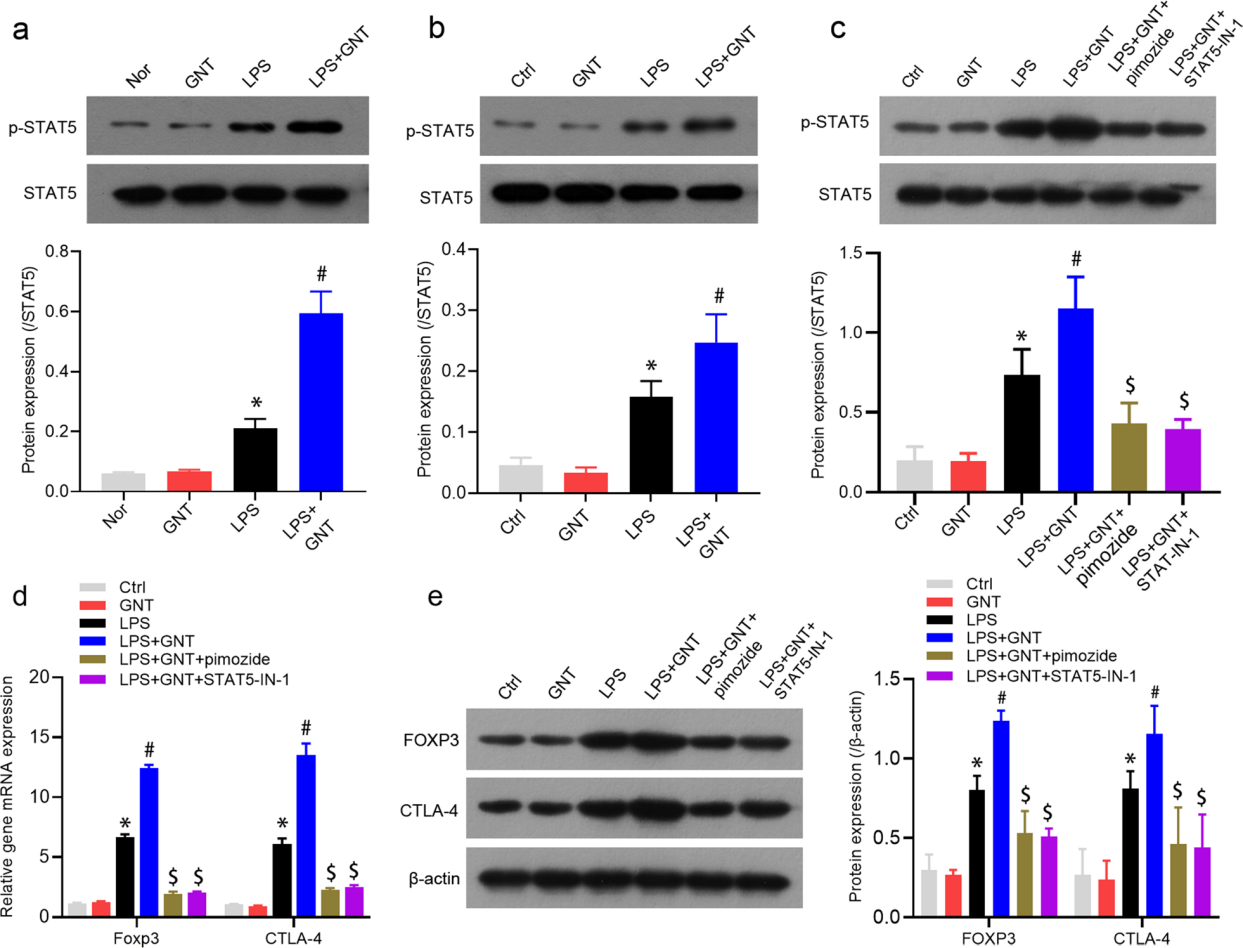
GNT modulated the phenotypic and functional changes of Tregs in vitro via activation of the STAT5 signaling pathway. **a** Western blot analysis of p-STAT5 protein expression levels in mice with LPS-induced neonatal sepsis treated with or without GNT in vivo. **b** p-STAT5 protein expression levels in the Tregs, which were isolated from normal mice and then stimulated with LPS and/or GNT in vitro. **c** p-STAT5 protein expression levels in the Tregs, which were isolated from normal mice and then treated with LPS, GNT, and/or STAT5 inhibitor pimozide or STAT5-IN-1 in vitro. **d** FOXP3 and CTLA-4 mRNA and protein expression levels in the Tregs, which were isolated from normal mice and then treated with LPS, GNT, and/or STAT5 inhibitor pimozide or STAT5-IN-1 in vitro. Data are expressed as mean ± SD, n = 3. *P < 0.05 versus control group; ^#^*P* < 0.05 versus LPS group; ^$^*P* < 0.05 versus LPS + GNT group

A recent study reported that STAT5 plays a crucial role in the development and function of Tregs [9]. To verify whether GNT regulates phenotypic and functional changes of Tregs via the STAT5 signaling pathway in vitro, we examined the effect of GNT on the STAT5 expression both in vitro and in vivo using Western blot. It was observed that p-STAT5 protein expression levels of Tregs were significantly increased in mice with LPS-induced sepsis compared to that with the control and GNT alone groups, which were further enhanced after GNT treatment (Fig. 5a). In addition, p-STAT5 protein expression levels in normal mice Tregs stimulated with LPS were significantly higher than that in normal mice Tregs stimulated with phosphate buffer saline (PBS) or GNT alone in vitro. This effect was further enhanced after GNT treatment (Fig. 5b). Next, p-STAT5 protein expression levels and FOXP3 and CTLA-4 mRNA and protein expression levels in normal mice Tregs treated with LPS, GNT, and/or STAT5 inhibitor pimozide or STAT5-IN were detected using qRT-PCR and Western blot. It was observed that pimozide suppressed p-STAT5 protein expression and FOXP3 and CTLA-4 mRNA and protein expressions in the LPS + GNT group (Fig. 5c–e).

### STAT5 inhibitor reversed GNT-induced the decreased secretion of inflammatory cytokines

**Fig. 6:**
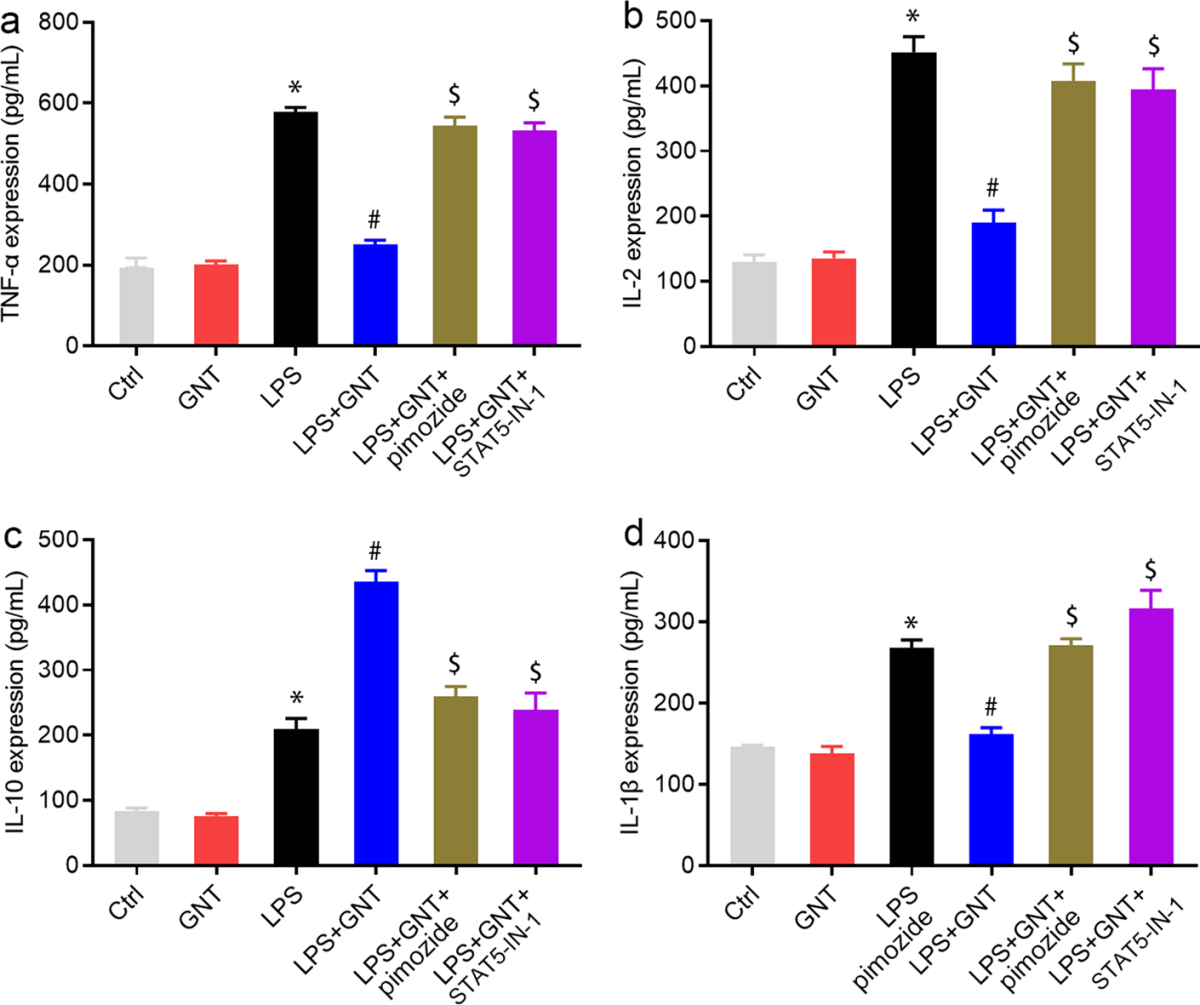
STAT5 inhibitor reversed GNT-induced the decreased secretion of inflammatory cytokines. After co-culture of CD4^+^CD25^−^ T cells and Tregs treated with anti-CD3 and anti-CD28 antibodies, cell culture supernatants were collected to examine TNF-α (**a**), IL-1β (**b**), IL-2 (**c**), and IL-10 (**d**) levels using ELISA. Data are expressed as mean ± SD, n = 3. *P < 0.05 versus control group; ^#^*P* < 0.05 versus LPS group; ^$^*P* < 0.05 versus LPS + GNT group

To further confirm the effect of GNT on Tregs, the expression levels of inflammatory cytokines were examined. Upon co-culture of CD4^+^CD25^−^ T cells and Tregs treated with anti-CD3 and anti-CD28 antibodies, LPS promoted the production of TNF-α, IL-2, IL-10, and IL-1β compared to that in the control and GNT alone groups. In addition, the concentrations of TNF-α, IL-2, and IL-1β decreased in the LPS + GNT group compared to that in the LPS group, whereas the concentrations of IL-10 increased in the LPS + GNT group. These effects were rescued by pimozide or STAT5-IN-1 treatment (Fig. 6, Additional file 3: Fig. S3).

### STAT5 inhibitor rescued the inhibitory effects of GNT on survival and inflammation in neonatal sepsis

**Fig. 7:**
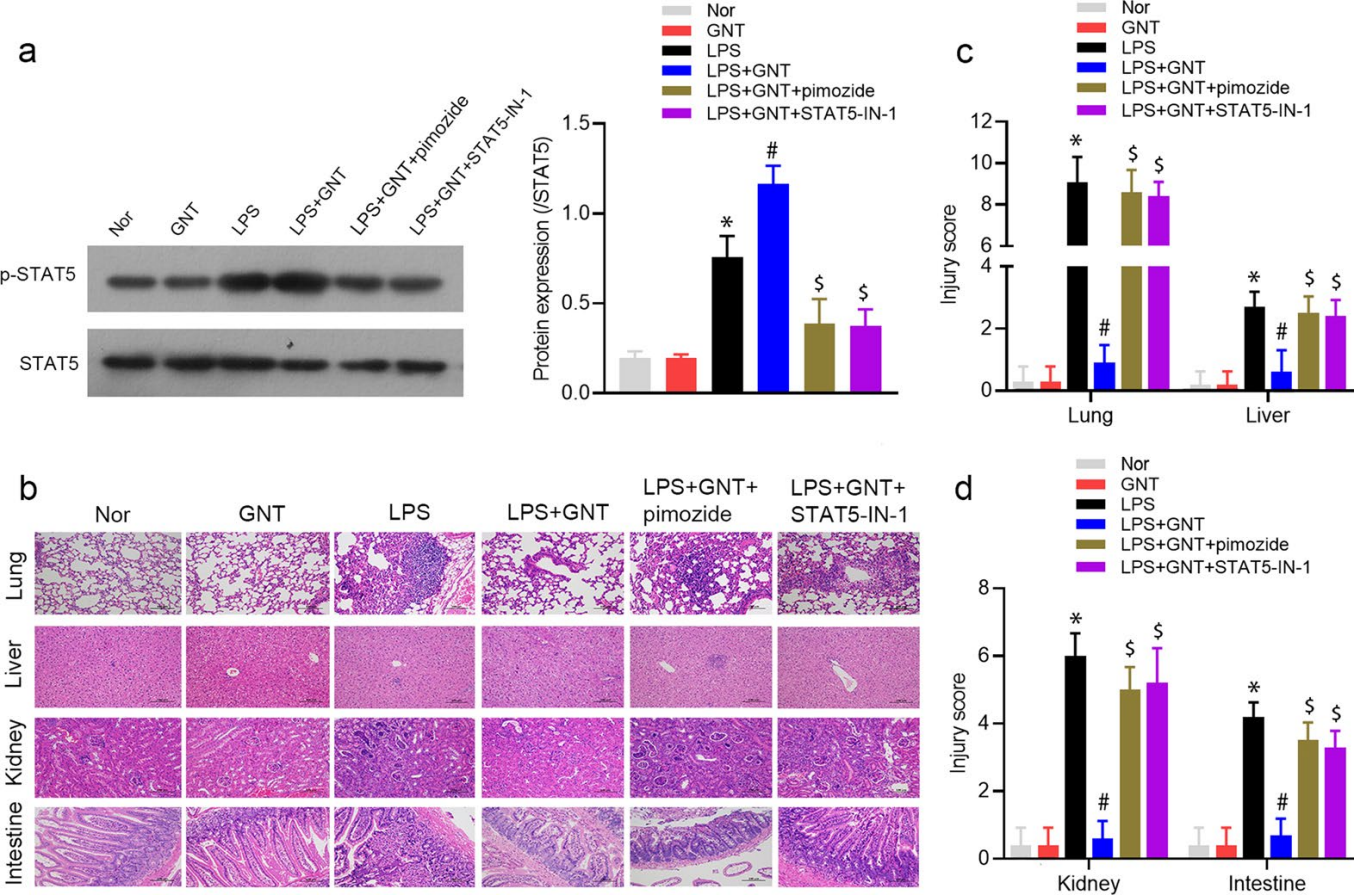
STAT5 inhibitor rescued the inhibitory effect of GNT on inflammation in neonatal sepsis. **a** The protein expression of p-STAT5 in LPS-induced mice treated with GNT and pimozide or STAT5-IN-1 was detected using western blot. Data are expressed as mean ± SD, n = 3. **b**, **c** HE staining of lung, liver, kidney, and intestine, and their injury scoring. Data are expressed as mean ± SD, n = 10. *P < 0.05 versus control group; ^#^*P* < 0.05 versus LPS group; ^$^*P* < 0.05 versus LPS + GNT group

**Fig. 8:**
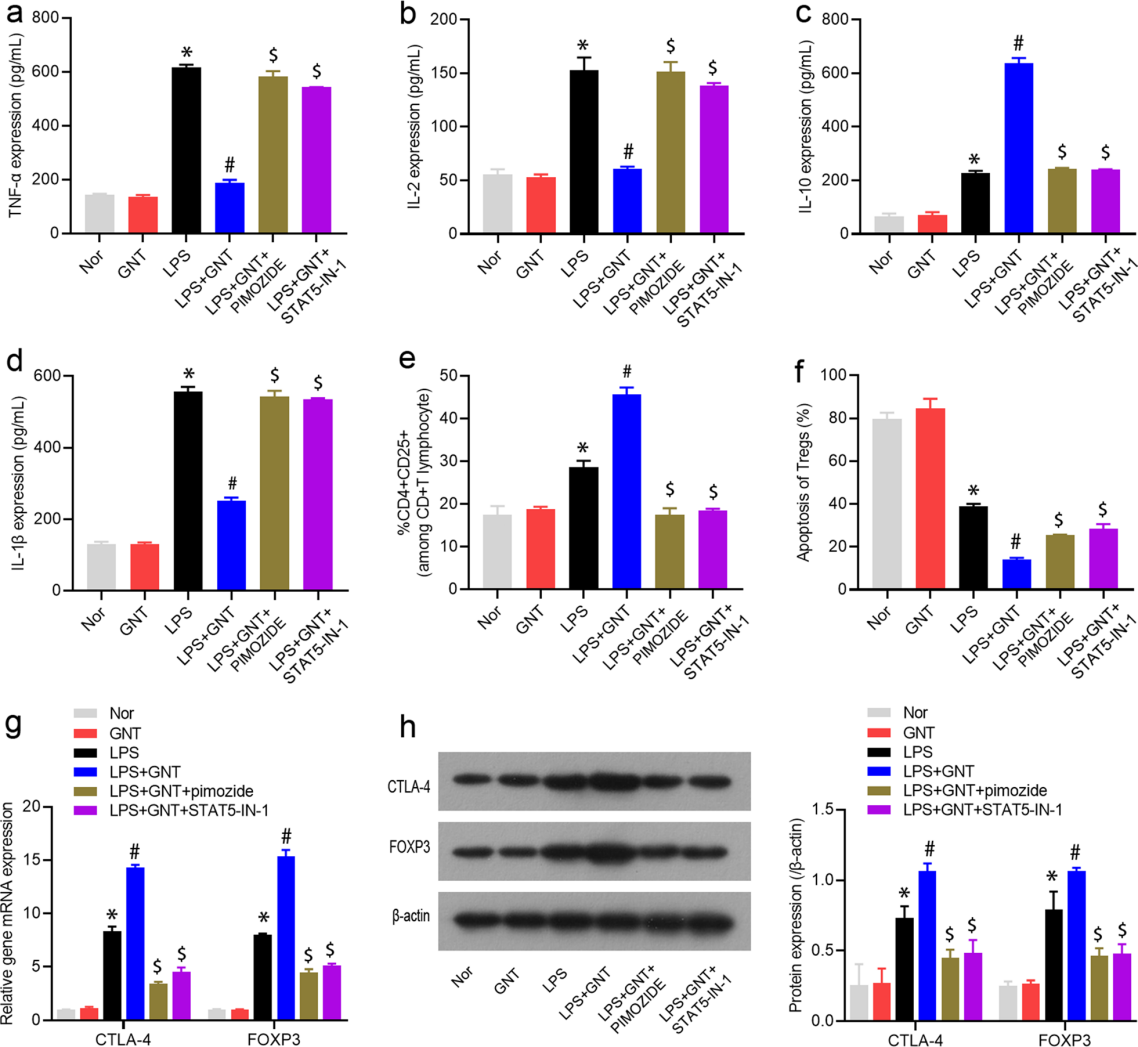
STAT5 inhibitor abrogated GNT-mediated impacts on the percentage, apoptosis, and phenotypic and functional changes of Tregs in neonatal sepsis. The protein levels of TNF-α (**a**), IL-1β (**b**), IL-2 (**c**), and IL-10 (**d**) in LPS-induced mice treated with GNT and pimozide or STAT5-IN-1. Data are expressed as mean ± SD, n = 10. Percentage (**e**) and apoptosis (**f**, **g**) of CD4 + Tregs in LPS-induced mice treated with GNT and pimozide or STAT5-IN-1. Data are expressed as mean ± SD, n = 10. **h** The mRNA expression levels of FOXP3 and CTLA-4. Data are expressed as mean ± SD, n = 10. **i** The protein expression levels of FOXP3 and CTLA-4. Data are expressed as mean ± SD, n = 3. *P < 0.05 versus control group; ^#^*P* < 0.05 versus LPS group; ^$^*P* < 0.05 versus LPS + GNT group

We further performed in vivo assays to validate GNT improved LPS-induced neonatal sepsis by activating STAT5 pathway. The survival and inflammation in neonatal sepsis were evaluated, and we observed that pimozide or STAT5-IN-1 treatment attenuated the upregulation of p-STAT5 protein expression induced by GNT and LPS treatment (Fig. 7a). The high survival rate of GNT + LPS group was also effectively restored by pimozide or STAT5-IN-1 treatment (Additional file 1: Fig. S1a). pimozide or STAT5-IN-1 treatment reversed the inhibitory effects on the histopathology changes of lungs, liver, kidneys, and intestines induced by GNT and LPS treatment (Fig. 7b, c). The increased IL-10 protein expression and decreased the protein expression of TNF-α, IL-2, and IL-1β in GNT + LPS group were rescued by pimozide or STAT5-IN-1 treatment (Fig. 8a–d).

### STAT5 inhibitor abrogated GNT-mediated impacts on the percentage, apoptosis, and phenotypic and functional changes of Tregs in neonatal sepsis

Finally, we used Flow cytometry, qRT-PCR and western blot analysis to explore whether GNT affects the STAT5 signaling pathway to regulate the percentage, apoptosis, and phenotypic and functional changes of Tregs in neonatal sepsis. As shown in Fig. 8e–g, Additional file 2: Fig. S2a, b, the reduced the percentage of Tregs and enhanced the apoptosis of Tregs induced by GNT and LPS treatment were restored by pimozide or STAT5-IN-1 treatment. Moreover, pimozide or STAT5-IN-1 treatment overturned the repressive effects on the levels of FOXP3 and CTLA-4 protein of Tregs mediated by GNT and LPS treatment (Fig. 8h, i).

## Discussion

GNT is an effective drug with anti-inflammatory and bactericidal properties for neonatal sepsis treatment [26, 27]. The present study demonstrated that GNT could mitigate inflammatory response and improve the survival outcome of neonatal sepsis by modulating the capacity of Tregs, an effect mediated via the STAT5 signaling pathway. Hence, GNT may increase the quantity and function of Tregs via activation of the STAT5 signaling pathway, attenuating inflammatory mediators in the pathogenesis of neonatal sepsis.

Neonatal sepsis causes morbidity and mortality in neonates [28], and sepsis-associated organ failure remains the most common cause of death [29]. Although efforts are being made in neonatal research to prevent sepsis progression, the pathogenesis of sepsis is complex, involving the innate and adaptive immune systems. The dysfunction of neutrophils, macrophages, and T lymphocytes prevents these cells from performing complete inflammatory responses in neonates. Tregs, which express a unique transcription factor of FOXP3, are vital in maintaining immune tolerance and sepsis balance [30]. A rising number of clinical and experimental studies have revealed that increased percentages and functions of Tregs are induced during the early stages of sepsis [31, 32], which is consistent with our results. In our study, increased percentage of CD4^+^ Tregs and expression of FOXP3 after LPS induction were observed in spleens of mice. However, whether this phenotype observed in the mesenteric lymph nodes need further investigation. In addition, GNT could upregulate Tregs and FOXP3 expressions in mice with neonatal sepsis and reduce the apoptotic rate of Tregs. GNT treatment significantly increased survival and alleviated inflammatory responses of lung, liver, kidney, and intestinal tissues with LPS-induced neonatal sepsis. The role of Tregs in sepsis remains controversial. Hotchkiss et al. reported that Tregs impair immunity and contribute to contribute to nosocomial infections and mortality [33]. However, several studies have shown that activated Tregs prevent polymicrobial sepsis mortality [32, 34], and regulate the activation status and antigen-presenting cell function and produce anti-inflammatory cytokines, which are beneficial to sepsis [35–37]. FOXP3 and CTLA-4 were used as biomarkers for defining Tregs function [38]. Our results suggested that GNT enhanced the function of Tregs, which manifested as increased FOXP3 and CTLA-4 mRNA and protein expression levels. Furthermore, GNT activated Tregs to reduce the mortality of LPS-induced mice, and decreased the concentrations of pro-inflammatory cytokines and increased the concentrations of anti-inflammatory cytokines, contributing to the recovery of neonatal sepsis. Additionally, Sun et al. showed that the pre-treatment of mice with GNT regulates the microbiota into guts and could impair the immune response to influenza virus infection [33]. However, whether the beneficial effects of GNT in sepsis were related to the reduction of bacterial translocation from guts and/or sepsis-related infection need further investigation.

Previous studies have reported that STAT5/CD4^+^CD25^+^FOXP3 Tregs pathway plays a critical role in the pathogenesis of chronic osteomyelitis [39, 40] and acute ischemic stroke [41]. In CD4^+^CD25^+^ Tregs, activation of STAT5 is necessary to sustain FOXP3 expression [42]. Moreover, the STAT5 signaling pathway increases the number of CD4^+^CD25^+^ FOXP3 Tregs [43]. In our study, STAT5 inhibitor suppressed the effect of GNT on p-STAT5, CTLA-4, and FOXP3 expression, and rescued the role of GNT in the concentrations of TNF-α, IL-2, IL-10, and IL-1β in CD4^+^ T lymphocytes. Furthermore, STAT5 inhibitor abrogated GNT-mediated impacts on survival and inflammation, and the percentage, apoptosis, and phenotypic and functional changes of Tregs in neonatal sepsis. These indicate that GNT exerts its protective effects in neonatal sepsis via activating the STAT5 signaling pathway to mediate the function of CD4^+^CD25^+^ Tregs.

## Conclusions

Our findings indicate that GNT could ameliorate inflammatory damage by modulating the quantity and function of Tregs via the STAT5 signaling pathway and lead us to new therapeutic strategies for GNT in neonatal sepsis.

## Supplementary Information

**Additional file 1: Fig. S1.** Percentage of CD4^+^ Tregs in different groups. **a** The survival rate of LPS-induced mice treated with GNT and pimozide or STAT5-IN-1. **b** Percentage of CD4^+^ Tregs in mice with LPS-induced neonatal sepsis treated with or without GNT were detected by Flow cytometry. Data are expressed as mean ± SD, n = 10. *P < 0.05 versus control group; ^#^*P* < 0.05 versus LPS group. ^$^*P* < 0.05 versus LPS+GNT group.

**Additional file 2: Fig. S2.** STAT5 inhibitor abrogated GNT-mediated impacts on the percentage and apoptosis of Tregs in neonatal sepsis. **a** Flow cytometry was used to examine the percentage of CD4^+^ Tregs in LPS-induced mice treated with GNT and pimozide or STAT5-IN-1. **b** Representative images of apoptosis.

**Additional file 3: Fig. S3.** The expression of TNF-α, IL-1β, IL-2, and IL-10 in the CD4^+^CD25^−^ T cells. After CD4^+^CD25^−^ T cells treated with anti-CD3 and anti-CD28 antibodies, cell culture supernatants were collected to examine TNF-α (**a**), IL-1β (**b**), IL-2 (**c**), and IL-10 (**d**) levels using ELISA. Data are expressed as mean ± SD, n = 3. *P < 0.05 versus control group; ^#^*P* < 0.05 versus LPS group; ^$^*P* < 0.05 versus LPS+GNT group.

## Data Availability

The datasets in this study are available from the corresponding author on reasonable request. The funding bodies had no role in the design of the study and collection, analysis, interpretation of data and writing of the manuscript.

## Abbreviations

GNT: Gentamicin
Tregs: Regulatory T cells
p-STAT5: Phosphorylation of signal transducer and activator of transcription 5
STAT5: The signal transducer and activator of transcription 5
LPS: Lipopolysaccharide
qRT-PCR: Quantitative real-time reverse transcription polymerase chain reaction
FOXP3: Forkhead helix transcription factor p3
CTLA-4: Cytotoxic T-lymphocyte-associated antigen 4
ELISA: Enzyme-linked immunosorbent assay
HE: Hematoxylin and eosin
