# Supplementary figures and images for: Gentamicin promoted the production of CD4+CD25+ Tregs via the STAT5 signaling pathway in mice sepsis
Source: BMC Immunol. 2022 Sep 26;23:47. doi: 10.1186/s12865-022-00521-4 (PMC9513864; doi:10.1186/s12865-022-00521-4)

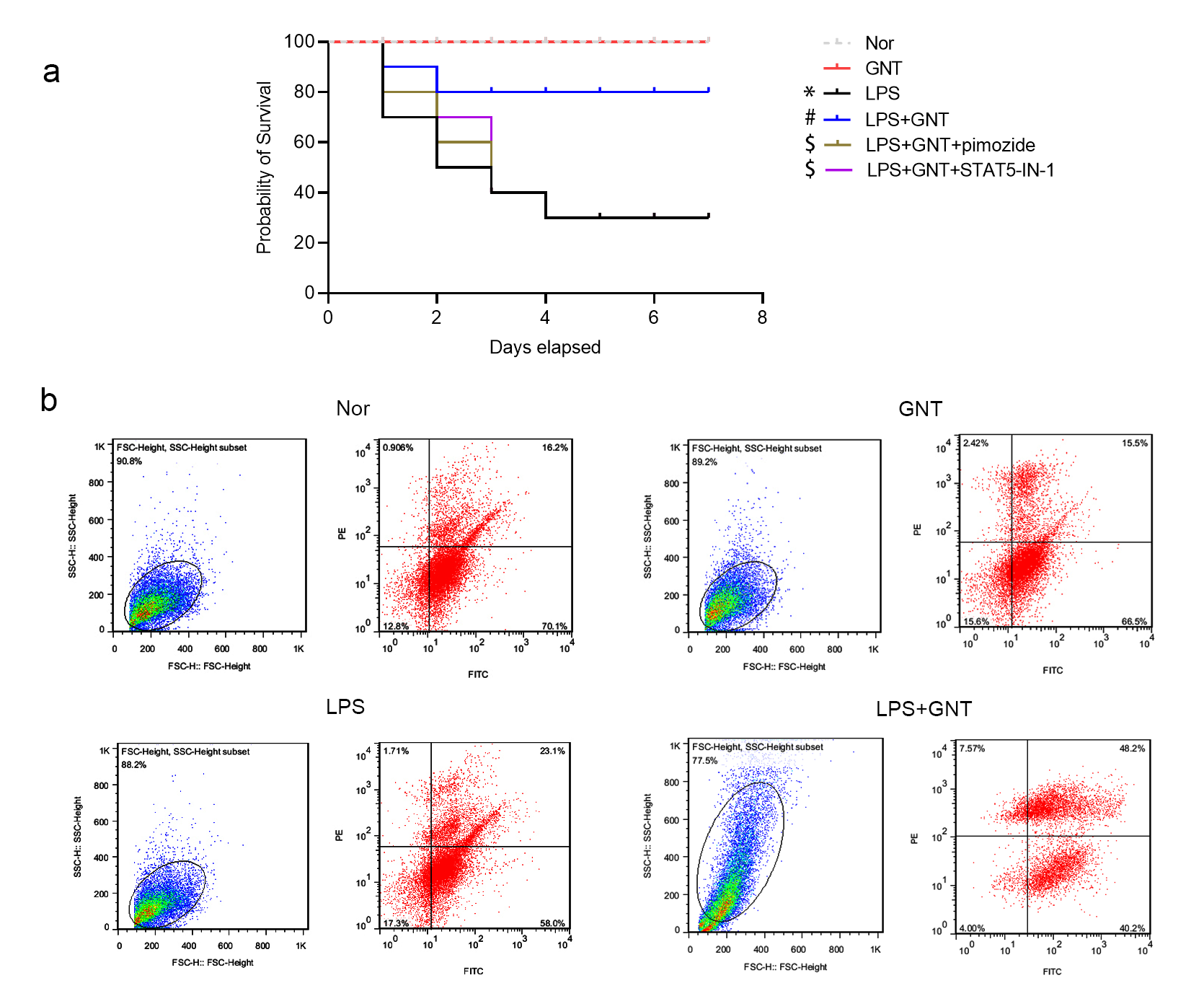

Supplement: Supplementary file 1 — Additional file 1: Fig. S1. Percentage of CD4+ Tregs in different groups. a The survival rate of LPS-induced mice treated with GNT and pimozide or STAT5-IN-1. b Percentage of CD4+ Tregs in mice with LPS-induced neonatal sepsis treated with or without GNT were detected by Flow cytometry. Data are expressed as mean ± SD, n = 10. *P < 0.05 versus control group; #P < 0.05 versus LPS group. $P < 0.05 versus LPS+GNT group. [file 12865_2022_521_MOESM1_ESM.tif]

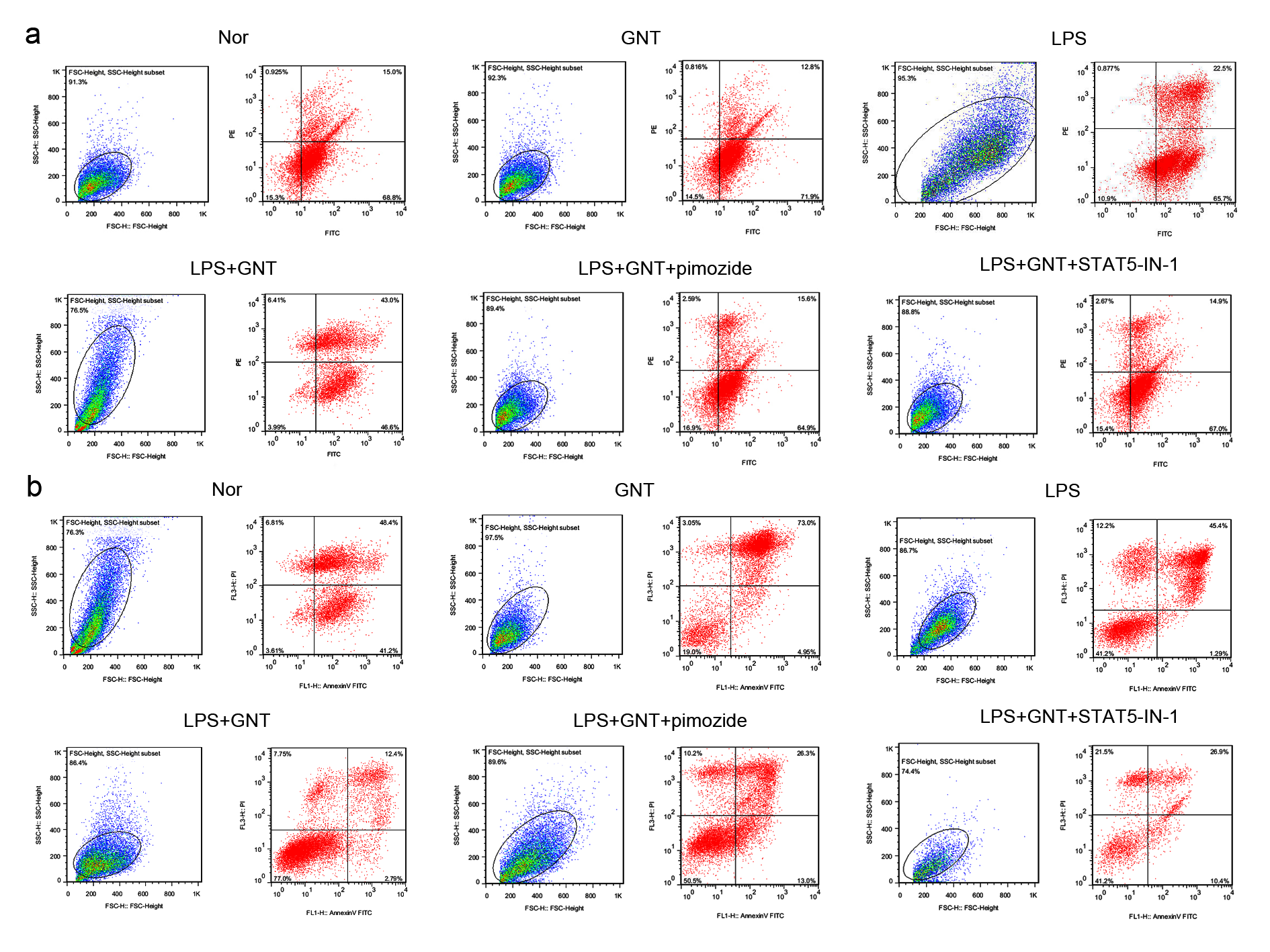

Supplement: Supplementary file 2 — Additional file 2: Fig. S2. STAT5 inhibitor abrogated GNT-mediated impacts on the percentage and apoptosis of Tregs in neonatal sepsis. a Flow cytometry was used to examine the percentage of CD4+ Tregs in LPS-induced mice treated with GNT and pimozide or STAT5-IN-1. b Representative images of apoptosis. [file 12865_2022_521_MOESM2_ESM.tif]

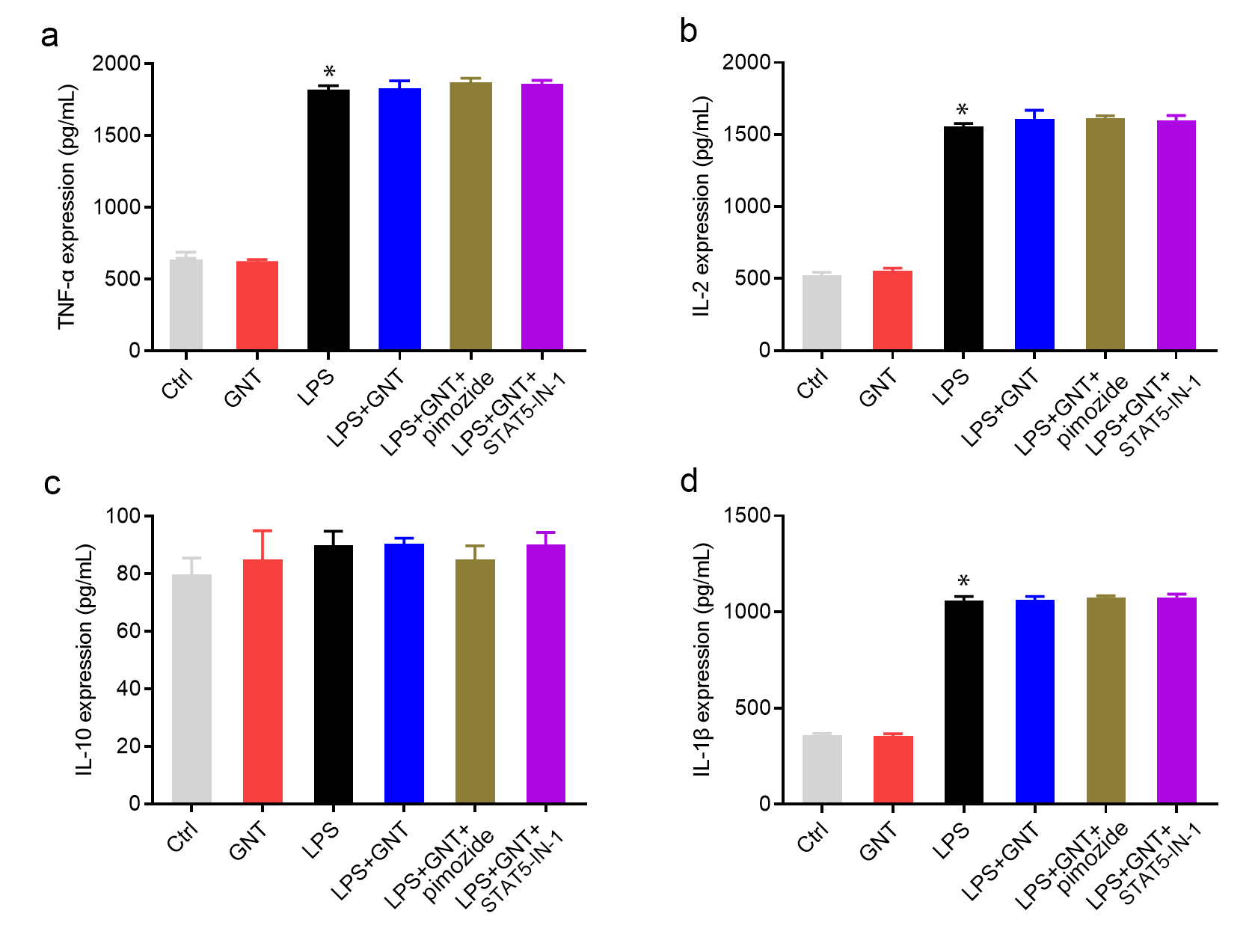

Supplement: Supplementary file 3 — Additional file 3: Fig. S3. The expression of TNF-α, IL-1β, IL-2, and IL-10 in the CD4+CD25− T cells. After CD4+CD25− T cells treated with anti-CD3 and anti-CD28 antibodies, cell culture supernatants were collected to examine TNF-α (a), IL-1β (b), IL-2 (c), and IL-10 (d) levels using ELISA. Data are expressed as mean ± SD, n = 3. *P < 0.05 versus control group; #P < 0.05 versus LPS group; $P < 0.05 versus LPS+GNT group. [file 12865_2022_521_MOESM3_ESM.tif]
